# Distinct Housing Conditions Reveal a Major Impact of Adaptive Immunity on the Course of Obesity-Induced Type 2 Diabetes

**DOI:** 10.3389/fimmu.2018.01069

**Published:** 2018-05-28

**Authors:** Julia Sbierski-Kind, Jonas Kath, Sebastian Brachs, Mathias Streitz, Matthias G. von Herrath, Anja A. Kühl, Katharina Schmidt-Bleek, Knut Mai, Joachim Spranger, Hans-Dieter Volk

**Affiliations:** ^1^Department of Endocrinology, Diabetes and Nutrition, Charité – Universitätsmedizin Berlin, Freie Universität Berlin, Humboldt-Universität zu Berlin, Berlin, Germany; ^2^Berlin Institute of Health (BIH), Berlin, Germany; ^3^Berlin-Brandenburg Center for Regenerative Therapies (BCRT), Charité – Universitätsmedizin Berlin, Freie Universität Berlin, Humboldt-Universität zu Berlin, Berlin, Germany; ^4^Partner Site Berlin, German Centre for Cardiovascular Research (DZHK), Berlin, Germany; ^5^Institute of Medical Immunology, Charité – Universitätsmedizin Berlin, Freie Universität Berlin, Humboldt-Universität zu Berlin, Berlin, Germany; ^6^Type 1 Diabetes Center, La Jolla Institute for Allergy and Immunology, La Jolla, CA, United States; ^7^iPATH Berlin – Core Unit Immunopathology for Experimental Models, Charité – Universitätsmedizin Berlin, Freie Universität Berlin, Humboldt-Universität zu Berlin, Berlin, Germany; ^8^Julius Wolff Institute (JWI), Center for Musculoskeletal Surgery, Charité – Universitätsmedizin Berlin, Freie Universität Berlin, Humboldt-Universität zu Berlin, Berlin, Germany

**Keywords:** obesity, adaptive immunity, type 2 diabetes, non-alcoholic steatohepatitis, housing conditions, insulin resistance

## Abstract

Obesity is associated with adipose tissue inflammation, insulin resistance, and the development of type 2 diabetes (T2D). However, our knowledge is mostly based on conventional murine models and promising preclinical studies rarely translated into successful therapies. There is a growing awareness of the limitations of studies in laboratory mice, housed in abnormally hygienic specific pathogen-free (SPF) conditions, as relevant aspects of the human immune system remain unappreciated. Here, we assessed the impact of housing conditions on adaptive immunity and metabolic disease processes during high-fat diet (HFD). We therefore compared diet-induced obesity in SPF mice with those housed in non-SPF, so-called “antigen exposed” (AE) conditions. Surprisingly, AE mice fed a HFD maintained increased insulin levels to compensate for insulin resistance, which was reflected in islet hyperplasia and improved glucose tolerance compared to SPF mice. By contrast, we observed higher proportions of effector/memory T cell subsets in blood and liver of HFD AE mice accompanied by the development of non-alcoholic steatohepatitis-like liver pathology. Thus, our data demonstrate the impact of housing conditions on metabolic alterations. Studies in AE mice, in which physiological microbial exposure was restored, could provide a tool for revealing therapeutic targets for immune-based interventions for T2D patients.

## Introduction

Type 2 diabetes (T2D) is a metabolic disease that is strongly associated with obesity and often preceded by insulin resistance. Obesity leads to adipose tissue dysfunction along with triglyceride accumulation, adipocyte hypertrophy, adipokine/cytokine secretion, hypoxia, endoplasmic reticulum stress, and impaired mitochondrial function, resulting in the activation of pro-inflammatory processes and adipose tissue fibrosis (1–4). Fibroblasts are also accumulated in injured regions from epithelial–mesenchymal transition and activated by Th2 immune responses (5). Hypoxia associated with adipocyte hypertrophy leads to the activation of NFκB-dependent pro-inflammatory responses in adipocytes and the expression of alarmin receptors. Necrotic adipocytes release cellular contents including alarmin, which has been shown to recruit immune cells and induce adipocyte death in turn (6, 7).

Chronic low-grade inflammation of hypertrophic adipose tissue plays an etiologic role in the development of insulin resistance (8). Obesity and obesity-related complications activate both the innate and adaptive arms of the immune system and lead to the recruitment of immune cells in metabolically active organs (9). For a long time, research focused on cells of the innate immune system, such as macrophages (10, 11), neutrophils (12), eosinophils (13), mast cells (14), natural killer cells (15), and dendritic cells (DCs) (16) infiltrating the visceral adipose tissue (VAT). However, recent data suggest the involvement of adaptive immunity, represented particularly by T cells (17, 18), showing the complexity of obesity-associated intratissue inflammation (19). Altogether, the causal impact of inflammation and immune responsiveness as well as the chronological order of inflammatory events remain largely unknown.

Currently, our understanding of the underlying mechanisms in these conditions is mainly based on experiments carried out in laboratory mice housed under specific pathogen-free (SPF) conditions. Those barrier facilities are unnaturally hygienic and do not reflect the microbial diversity humans encounter in their environment. Despite success in efficient translation of animal studies to the clinic, many murine models failed to translate promising treatments of disease models to clinical studies, as relevant aspects of the human immune system are unappreciated by SPF mice (20, 21). The association between chronic inflammation caused by activation of pro-inflammatory targets and cytokines and obesity suggest that long-term blockade of those cytokines may be used to treat or prevent obesity-induced inflammation. However, even if preclinical studies in rodent models of obesity have been promising in using such strategies, clinical trials in humans have had mixed success. In a recent type 1 diabetes, prevention trial oral insulin was given to prediabetic patients based on evidence from animal models which showed that oral insulin could induce anti-inflammatory immune activation to suppress anti-islet responses, however, the delayed disease progression was observed only in a subgroup of patients (22). Furthermore, another immune-specific clinical trial investigating effects of Etanercept in patients with metabolic syndrome failed due to such translational difficulties (23).

Recent studies claimed that murine models housed under SPF conditions lack effector/memory T cells in contrast to mice exposed to antigens, such as pet shop and free-living mice (23–25). Thus, chronic exposure to antigens from environmental germs under “natural conditions” results in reversed proportions of naïve and effector/memory T cells and a more restricted T cell receptor repertoire, associated with changes in the B cell and myeloid compartment. This process is called “aging” of the adaptive immune system, indicated by increased frequencies of effector/memory T cells in blood and immune organs. In contrast to naïve and early memory T cells, effector/memory T cells express an altered homing and stimulation pattern enabling them to infiltrate inflamed tissue sites and contribute to an intratissue inflammatory state with major impact on tissue homeostasis. Recently, we could establish a model of antigen exposure under standardized non-SPF housing conditions (23). Applying this model, we investigated the impact of adaptive immunity over the course of obesity-related T2D. For this purpose, we analyzed the immune cell composition in blood, adipose tissue, and liver as well as markers of glucose intolerance and insulin secretion in SPF and non-SPF mice [called “antigen exposed” (AE), here], receiving normal diet (ND) or high-fat diet (HFD).

## Materials and Methods

### Animals and Diet

This study was carried out in accordance with the Guide for the Care and Use of Laboratory Animals of the National Institutes of Health and the Animal Welfare Act under the supervision of our institutional Animal Care and Use Committee. Animal protocols were conducted according to institutional ethical guidelines of the Charité Berlin, Germany, and were approved by the Landesamt für Gesundheit und Soziales (approval number G 0138/14, LAGeSo Berlin, Germany) and comply with the ARRIVE guidelines.

Mice were housed in SPF conditions or transferred to the AE animal facility at the age of 4 weeks. Table S1 in Supplementary Material provides an overview of the specific SPF and AE housing conditions. AE housing was complemented with daily non-specific microbial exposure to antigens from bedding of mammalian laboratory animals (sheep and pigs) housed in rooms next to the laboratory mice. The exposure was guaranteed by daily handling of the laboratory animals and antigens were distributed by air passage, as the laboratory staff switched rooms on a regular basis. A HFD (60 kJ% from fat, 19 kJ% from proteins, and 21 kJ% from carbohydrates, SSNIFF, E15741-34, Soest, Germany) for mice was started at 5 weeks of age and continued for either 7 or 15 weeks. Age-matched animals were maintained on ND (9 kJ% from fat, 33 kJ% from proteins, and 58 kJ% from carbohydrates, SSNIFF, V1534-300, Soest, Germany). Body weight was followed weekly throughout the course of the experiment.

### Analysis of Metabolic Parameters

Intraperitoneal glucose tolerance test (IPGTT) was performed in overnight fasted mice by injecting glucose (1 g/kg body weight; B. Braun Melsungen, Melsungen, Germany) and blood glucose levels were monitored at 0, 15, 30, 60, and 120 min postglucose administration and plotted against time curves to determine the glucose tolerance. Serum insulin was measured by ELISA kit using a rat insulin standard (Crystal Chem, Downers Grove, IL, USA). Body composition was determined by nuclear magnetic resonance using a Bruker Minispec instrument (Bruker, Woodlands, TX, USA). Real-time metabolic analyses were conducted using a combined indirect calorimetry system (TSE Systems, GmbH, Bad Homburg, Germany) as previously described (26).

### Histological Analysis

Following perfusion with saline to minimize contamination with cells from the vasculature, liver, and pancreatic tissue were dissected from the surrounding tissues, fixed in formalin, and embedded in paraffin. The sections (4 µm) were deparaffinized with xylene and rehydrated through an ethanol gradient.

For immunohistochemical (IHC) staining in pancreatic sections antigen was retrieved by citric acid buffer (pH 6.0) and slides were blocked with normal goat serum and incubated with anti-insulin antibody (Abcam, Cambridge, UK, 1:200) overnight at 4°C. Slides were then washed and incubated with an HRP-conjugated secondary antibody (Abcam, Cambrige, UK) for 1 h at room temperature. After washing in PBS, HRP was visualized by NovaRED (VECTOR NovaRED Peroxidase Substrate Kit, Vectorlabs, Peterborough, UK) and nuclei were counterstained with hematoxylin. Islets were marked by insulin-positive β cells. The average islet area and number of islets were calculated per total pancreatic area in a minimum of five sections per sample, 200 µm apart.

Liver was processed for routine hematoxylin/eosin (HE) and histochemical staining. Tissue collagen deposition was detected by applying the following histochemical staining protocol: slides were incubated with a 0.1% Sirius Red solution dissolved in saturated picric acid for 1 h washed in acidified water (0.5% hydrogen chloride), dehydrated, and mounted with DPX mounting. Collagen and non-collagen components were red- and orange-stained. For IHC staining, antigens were unmasked by boiling in citrate buffer for 30 min. Slides were blocked using 0.3% H_2_O_2_/H_2_O for 10 min and incubated overnight with purified monoclonal rat IgG2b kappa anti-mouse I-A/I-E (BioLegend, Germany, 1:50). Biotinpolyclonal goat anti-rat (1:50) was used as second antibody. Slides were then incubated with Avidin-HRP (Thermo Fisher Scientific, Germany, 1:2,000) for 30 min. HRP was detected with NovaRed (VECTOR NovaRED Peroxidase Substrate Kit, Vectorlabs, Peterborough, UK) and slides were counterstained with HE.

The slides were analyzed with a Zeiss Axioplan light microscope (Carl Zeiss, Germany) and the images were acquired using AxioVert.

### Tissue Cell Preparation

Mice were sacrificed under anesthesia. For isolation of stromal vascular cells, epididymal adipose tissues were removed, minced into small pieces and then incubated in a rotational shaker (200 rpm) at 37°C for 20 min in collagenase solution (1 mg/ml collagenase type 1, Worthington, UK, with 0.5% BSA and 10 mM CaCl_2_ in PBS). The digested tissue was passed through a 100-µm mesh and centrifuged (500 *g*, 10 min). The pellet containing the stromal vascular fraction was resuspended with erythrocyte lysis buffer (Sigma Aldrich, Germany) and centrifuged (500 *g*, 5 min). Cells were washed again in 2% FCS in PBS, resuspended in 250 µl 2% FBS in PBS, and counted *via* trypan blue exclusion. Livers were perfused with PBS and minced. Liver mononuclear cells were obtained by collagenase digestion of liver tissue for 30 min at 37°C on a rotation shaker (200 rpm). Following digestion, the cells were centrifuged (30 *g*, 1 min, RT) to pellet hepatocytes. Cells in the supernatant were then centrifuged at (310 *g*, 4 min, 4°C) and resuspended in 30% Percoll solution in HBSS followed by centrifugation (800 *g*, 30 min, RT) to enrich liver mononuclear cells. Erythrocytes were lysed in 8 ml erythrocyte lysis buffer (Sigma Aldrich, Germany) and 2 ml HBSS and centrifuged (300 *g*, 10 min, 4°C). Cell viability was checked with trypan blue exclusion.

### Flow Cytometry

Isolated cells were incubated for FC blocking with CD16/32 antibody (BioLegend, Germany) for 10 min and antibodies for 20 min, washed twice, and filtered through a 35-µm mesh. Antibodies for surface staining are listed in Table S3 in Supplementary Material. FACS analysis was performed on a BD-LSR Fortessa. FACS data were analyzed by post collection compensation using FlowJo 10.0.8 software (Tree Star, Ashland, OR, USA).

### Participants

Blood samples were collected from a total of 21 obese women (body mass index ± SD: 34.61 ± 3.76 kg/m^2^) included in a study focusing on muscle mass regulation “Effects of negative energy balance on muscle mass regulation” (registered at https://clinicaltrials.gov, NCT01105143) at the Department of Endocrinology, Charité, Berlin.

This study was carried out in accordance with the recommendations of the International Conference on Harmonization Guidelines for Good Clinical Practice and the Declaration of Helsinki. The protocol of the study was approved by the local Ethics Committee of the Charité (EA2/050/10). All subjects gave written informed consent in accordance with the Declaration of Helsinki before participating in this study.

### Human Antibody Panel

Fluorochrome-conjugated anti-human monoclonal antibodies were obtained from Beckman Coulter (Marseille, France) and anti-BDCA-2 and anti-BDCA-3 from Miltenyi Biotec (Bergisch Gladbach, Germany). Six panel matrices for 7- to 9-fluorochrome channels defined and validated by the ONE Study consortium were used based on published results (27).

### Human Leukocyte Staining

100 µl of anticoagulated peripheral blood cells was stained with surface antibodies as previously described (27). All samples were measured on 10 color, 3 laser Navios flow cytometers (Beckman Coulter), and acquired data were analyzed using the Kaluza software, version 1.2 (Beckman Coulter).

### Statistical Analysis

Results are shown as the mean ± SEM. All analyses were performed using GraphPad Prism, version 6 (GraphPad Software, San Diego, CA, USA). *P* values <0.05 were considered significant. Depending on data distribution, Pearson simple coefficient or Spearman rank correlation coefficient with two-tailed significance test were used for correlation analysis, and Mann–Whitney *U*-test, Student’s *t* test, or one-way ANOVA followed by Tukey’s comparison test were applied to estimate differences between groups as appropriate. The body weight, glucose, and insulin time course data were analyzed by repeated measurements two-way ANOVA followed by Bonferroni *post hoc* test.

## Results

### SPF Mice Lack Effector Memory T Cell Subsets

**Figure 1:**
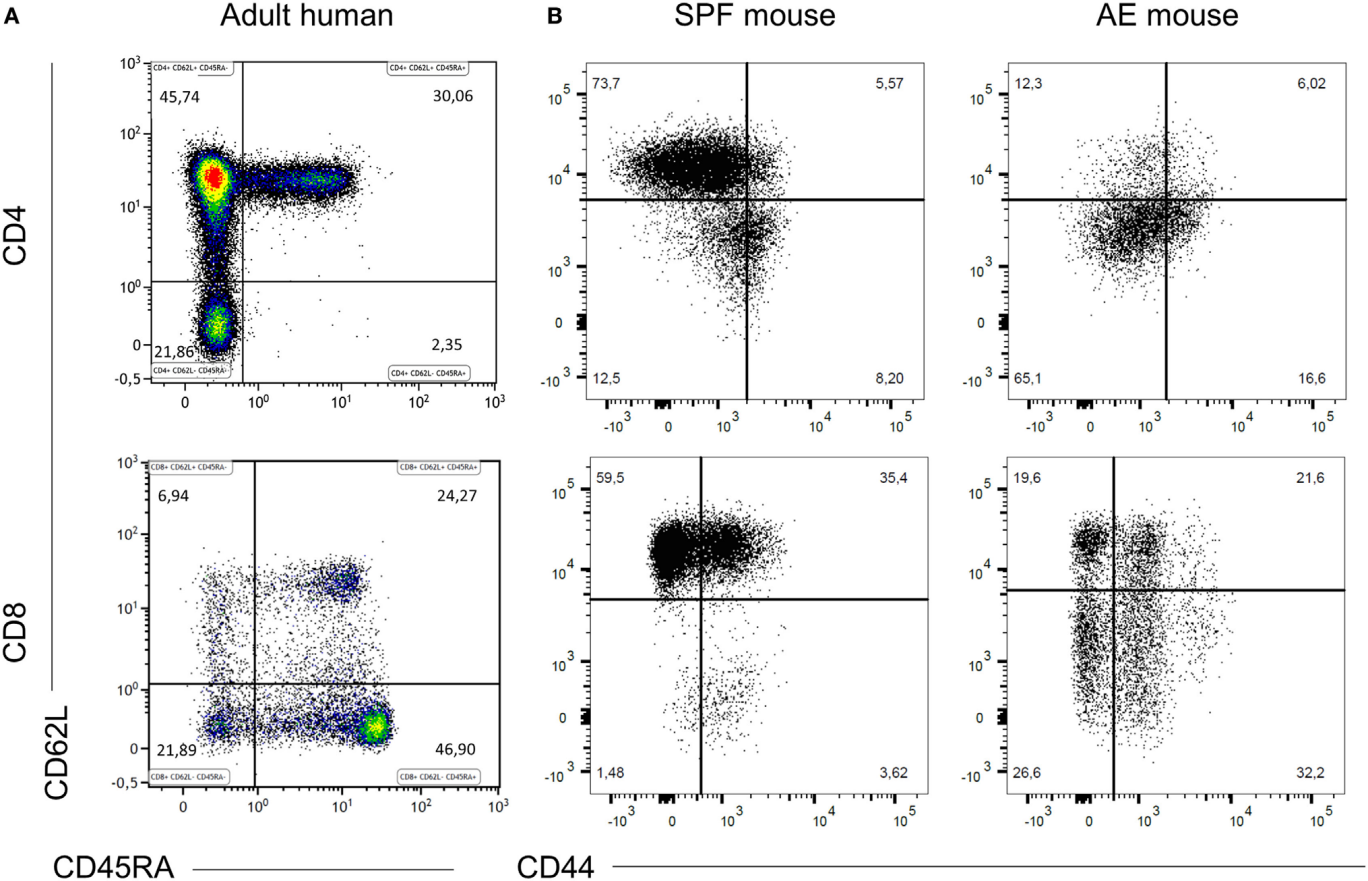
Specific pathogen-free (SPF) mice lack effector memory T cell subsets. CD4^+^ and CD8^+^ T cell phenotypes were compared between 20 weeks old SPF mouse blood (*n* = 10), 20 weeks old antigen-exposed (AE) mouse blood (*n* = 10), and adult human blood (*n* = 21) by fluorescence flow cytometry. Top panels are gated on CD3^+^CD4^+^ cells and bottom panels are gated on CD3^+^CD8^+^ cells and show naïve (CD44^−^CD62L^+^/CD45RA^+^CD62L^+^), central memory (CD44^+^CD62L^+^/CD45RA^−^CD62L^+^), effector memory (CD44^+^CD62L^−^/CD45RA^−^CD62L^−^), and effector (CD44^−^CD62L^−^/CD45RA^+^CD62L^−^) T cells. **(A)** Representative dot plots of blood T cell subset distribution in adult human. **(B)** Representative dot plots of blood T cell subset distribution in SPF and AE mouse. Numbers in panels indicate percentages of T cell subsets.

To visualize differences in immune responses, we compared T cell distribution between SPF, AE mice, and humans. Relative to adult humans (Figure 1A), SPF mice showed lower frequencies of effector memory (CD44^+^CD62L^−^) T cells (<30%), near absence of effector (CD44^−^CD62L^−^) T cells (<5–10%), and reciprocally, higher frequencies of naïve (CD44^−^CD62L^+^) T cells (>60%) in blood, particularly in the CD8^+^ T cell compartment (Figure 1B). By contrast, decreased proportions of naïve T cells (<40%) and enhanced levels of effector memory T cells (>25%) were observed in AE mice, which appeared much closer to the human phenotype (Figure 1B).

### Altered Immune Cell Composition in AE Mice Kept on HFD

**Figure 2:**
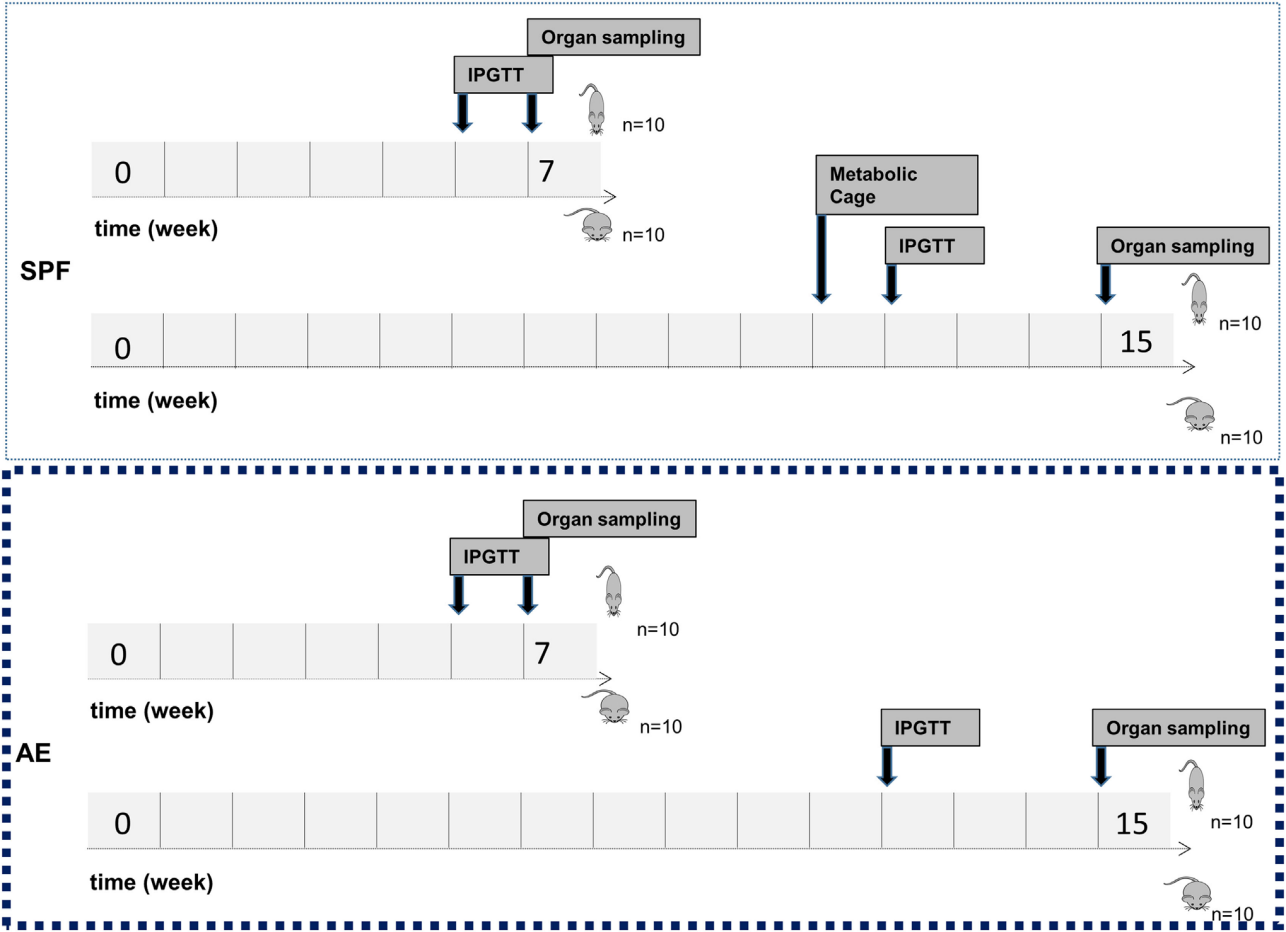
Study flowchart. Mice were kept in specific pathogen-free (SPF) conditions or transferred to the non-SPF animal facility at the age of 4 weeks. At the age of 6 weeks, male C57Bl/6J mice were fed either a normal diet (ND) or a high-fat diet (HFD) for either 7 or 15 weeks with 10 mice per group. Intraperitoneal glucose tolerance test (IPGTT) was performed after 6 or 12 weeks of ND or HFD feeding. Metabolic phenotyping was performed after 11 weeks of ND or HFD feeding. Animals were sacrificed at the age of 12 or 20 weeks for sample collection.

**Figure 3:**
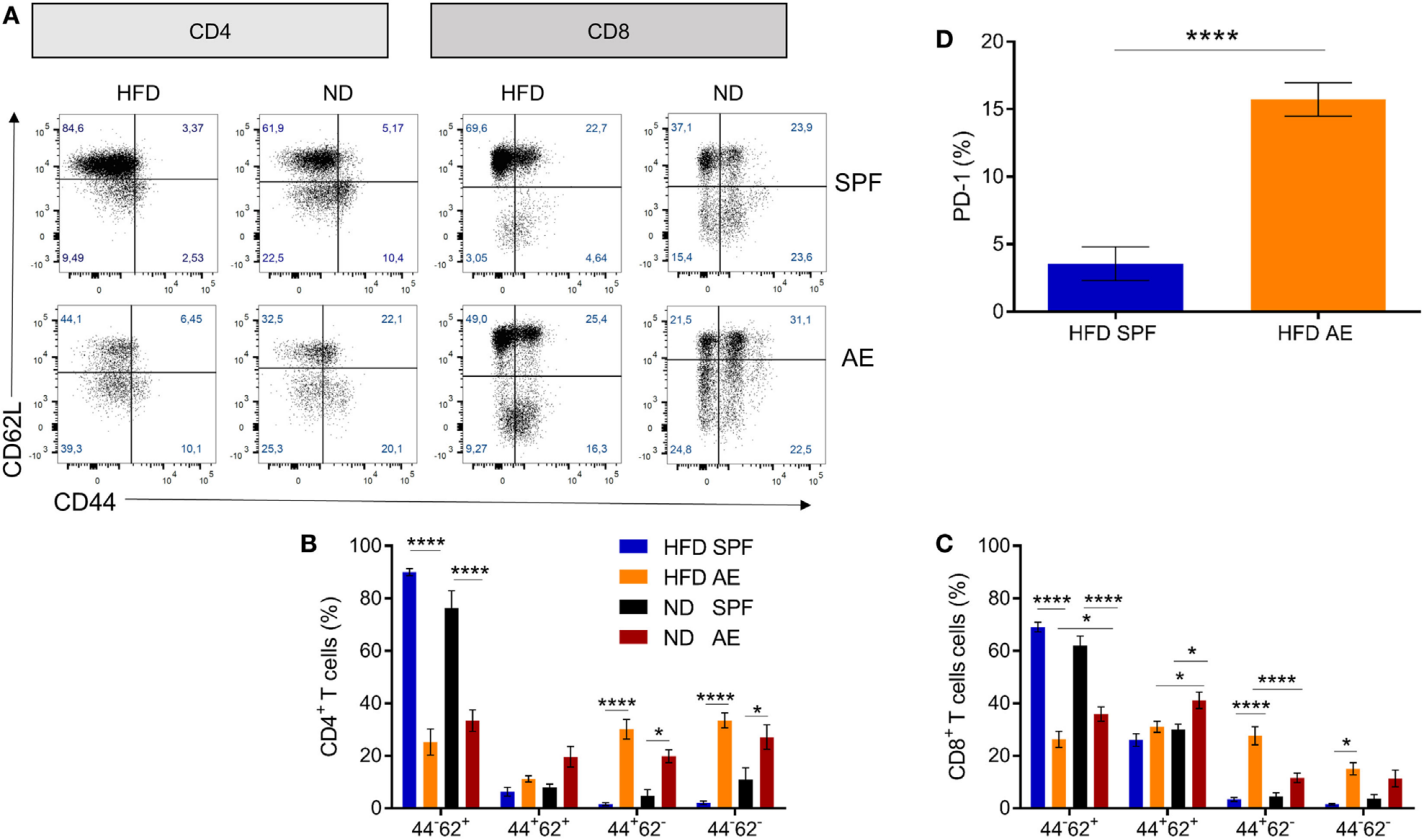
Altered immune cell composition in antigen-exposed (AE) mice kept on high-fat diet (HFD). **(A)** Representative dot plots for CD4^+^ and CD8^+^ T cell subsets in blood from specific pathogen-free (SPF) and AE mice fed a HFD or normal diet (ND) for 7 weeks. **(B,C)** Percentages of naïve (CD44^−^CD62^+^), central memory (CD44^+^CD62L^+^), effector memory (CD44^+^CD62L^−^), and effector (CD44^−^CD62L^−^) CD4^+^ and CD8^+^ T cells. Data are represented as mean ± SE. Significance was determined using two-way ANOVA multiple measurement. **(D)** PD-1 expression of CD44^+^CD62L^−^ T cells in HFD SPF compared to AE mice. Data are represented as mean + SE. Significance was determined using unpaired two-sided Mann–Whitney *U*-test. *n* = 10 mice per group. *P < 0.05, ****P < 0.0001.

To assess the combined impact of housing conditions and diet on the immune composition as well as on the metabolic status, we compared AE and SPF mice on HFD and ND. The experimental design is shown in Figure 2. All groups were metabolically characterized and blood was analyzed either at week 7 or 15. Similar to previous observations, blood from AE mice was enriched in effector memory T cells and, correspondingly, showed lower percentages of naïve T cells (both CD4^+^ and CD8^+^) compared to SPF mice (Figures 3A–C). In addition, a subset of CD44^−^CD62L^−^ T cells strongly increased under AE conditions as an equivalent to effector T cells (T_emra_) in humans. Remarkably, the frequency of effector memory T cells increased further under HFD in AE mice, however, not in SPF mice (Figures 3B,C). The significantly enhanced expression of PD-1 (CD 279), associated with chronic T cell activation, in AE mice matched these data (Figure 3D). Furthermore, the total amount of leukocytes and T cells was higher in AE compared to SPF mice (data not shown). Taken together, housing conditions and diet have a major impact on T cell subset distributions.

### Preserved Pancreatic β Cell Responsiveness in HFD AE Mice

**Figure 4:**
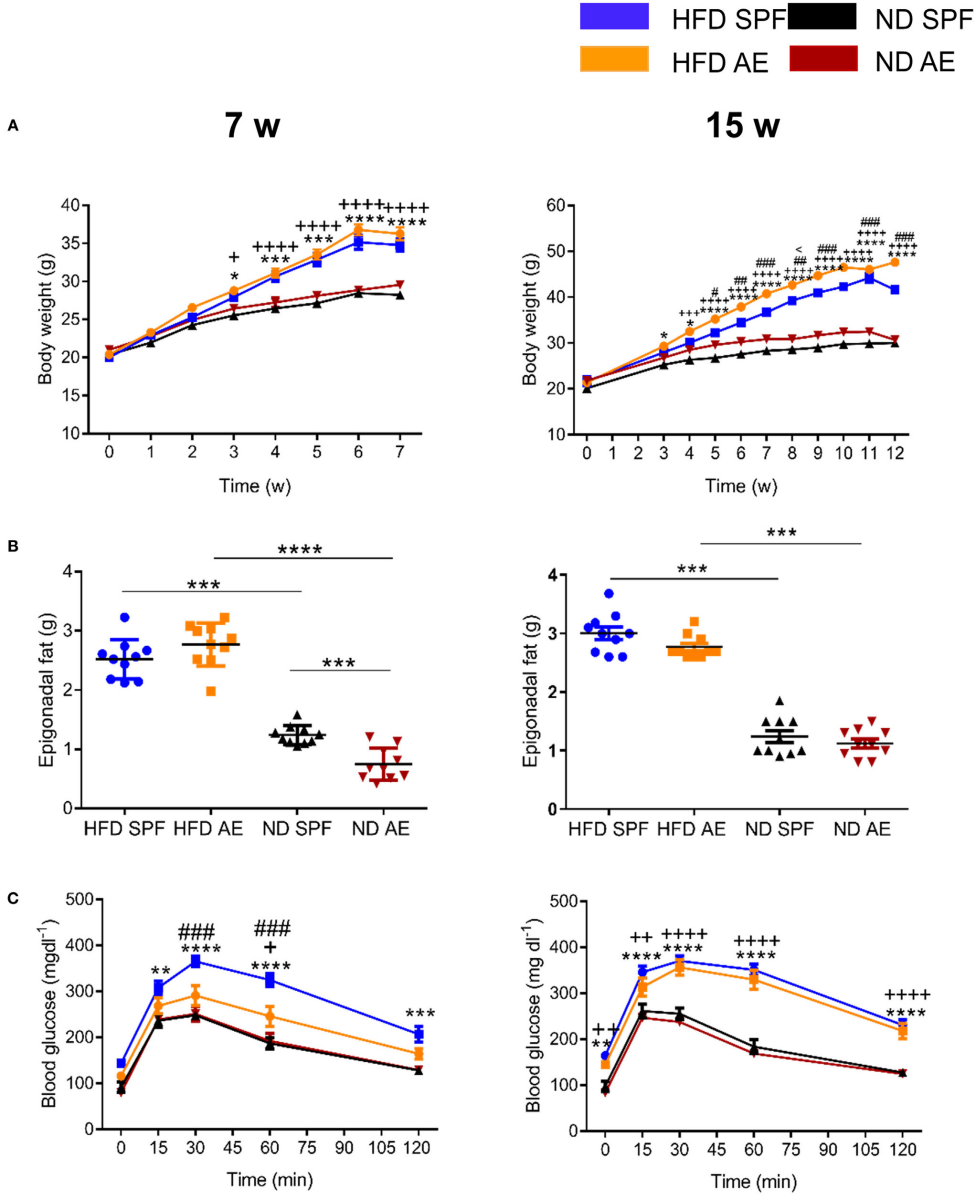

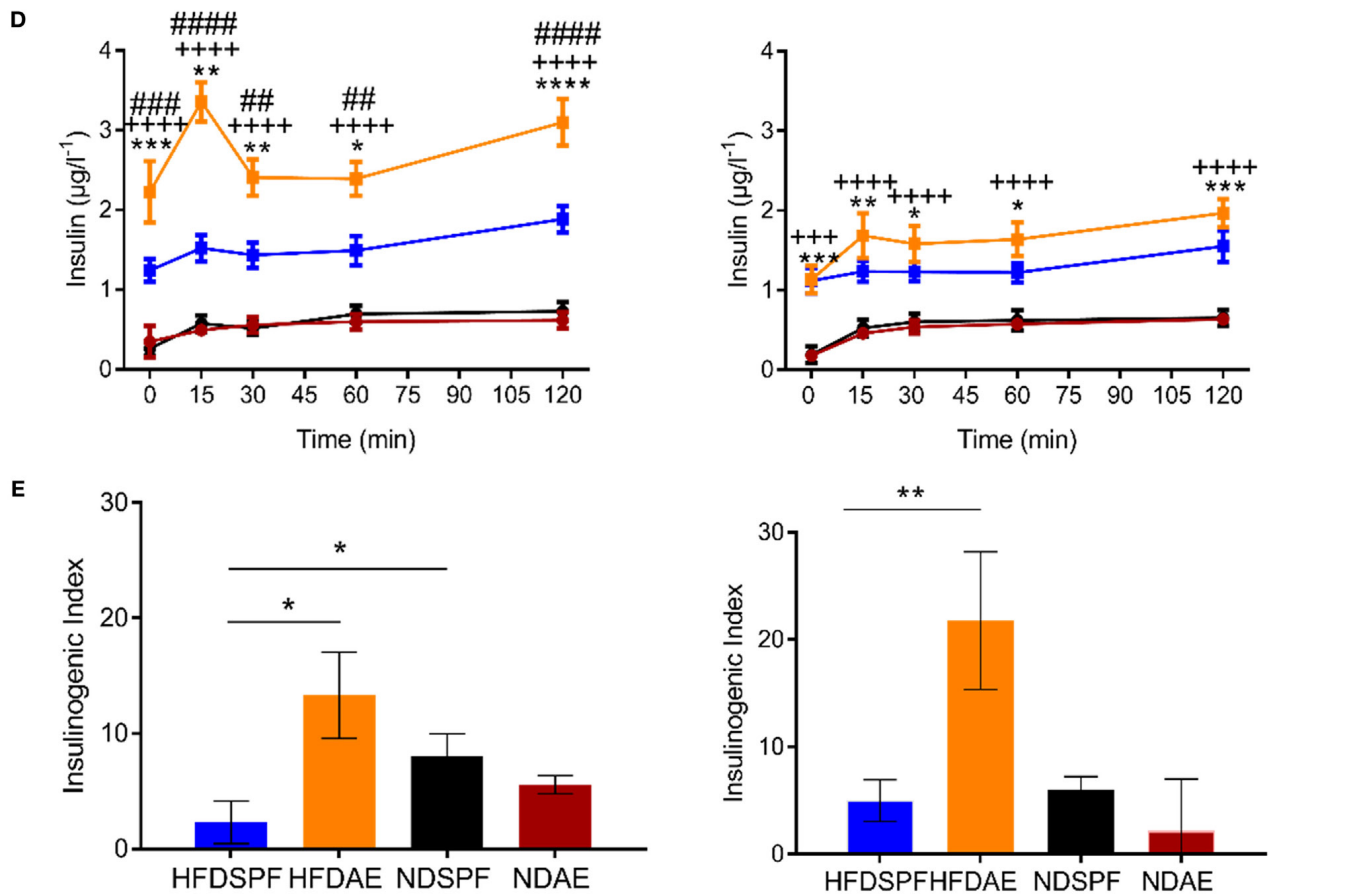
Preserved pancreatic β cell responsiveness in antigen-exposed (AE) high-fat diet (HFD) mice. **(A)** Weight development in 7-week (w, left) and 15-week (right) normal diet (ND) and HFD specific pathogen-free (SPF) vs. AE mice. **(B)** Weight of epididymal fat pads. **(C)** Intraperitoneal glucose tolerance tests were performed in ND and HFD SPF vs. AE mice after 7 weeks (left) and 15 weeks (right) feeding. **(D)** Circulating insulin levels were assessed before and after intraperitoneal glucose injection in ND and HFD SPF vs. AE mice after 7 weeks (left) and 15 weeks (right) feeding. **(E)** Calculated insulinogenic index, *n* = 10 mice per group. Significance was determined using two-way ANOVA multiple measurement **(A,C,D)** or using unpaired two-sided Mann–Whitney *U*-test. **(B,E)** *P < 0.05, **P < 0.01, ***P < 0.001, ****P < 0.0001. *(HFD SPF vs. ND SPF), ^++^(HFD AE vs. ND AE), ^#^(HFD SPF vs. HFD AE), ^<^(ND SPF vs. ND AE).

To assess the impact of “aged” adaptive immunity following antigen exposure on the development of insulin resistance and glucose intolerance, we examined the metabolic effects of HFD long-term feeding in SPF and AE mice. HFD feeding resulted in the development of metabolic alterations already after 7 weeks in SPF mice, as shown in Figures S1A–G in Supplementary Material. Within both cohorts (SPF and AE), a significant difference in body weight between HFD and ND mice was first evident at week 4 (*P* < 0.001) and remained significant throughout the following experimental weeks (Figure 4A). HFD-fed AE mice tended to gain more weight after 7 weeks and were even significantly heavier than their SPF counterparts after 12 weeks of HFD feeding. By contrast, epididymal fat pads were enlarged in HFD mice but remained unaffected by housing conditions (Figure 4B). Whereas glucose tolerance was impaired in HFD mice maintained under SPF conditions, surprisingly, the glucose levels of HFD AE mice were much lower and did not differ significantly from those of the ND AE and SPF mice after 7 weeks (Figure 4C). This striking difference was not observed at a later time point (15 weeks) in HFD-fed mice although fasting and peak glucose levels remained slightly lower in AE compared to SPF mice (Figure 4C). Insulin levels, measured throughout the IPGTT, were significantly higher in HFD AE mice (Figure 4D). The insulinogenic index, the ratio of the increment in insulin concentration to the increment in glucose concentration (ΔI/ΔG) has been proposed as a measure for β cell function and is highest in HFD-fed AE mice at all time points (Figure 4E). In summary, HFD AE mice develop obesity and insulin resistance but show adequate β cell compensation and a delayed development of diabetes in contrast to HFD SPF mice.

### Diet-Induced Obesity Rather Than Housing Conditions Change the Immune Cell Composition in VAT

**Figure 5:**
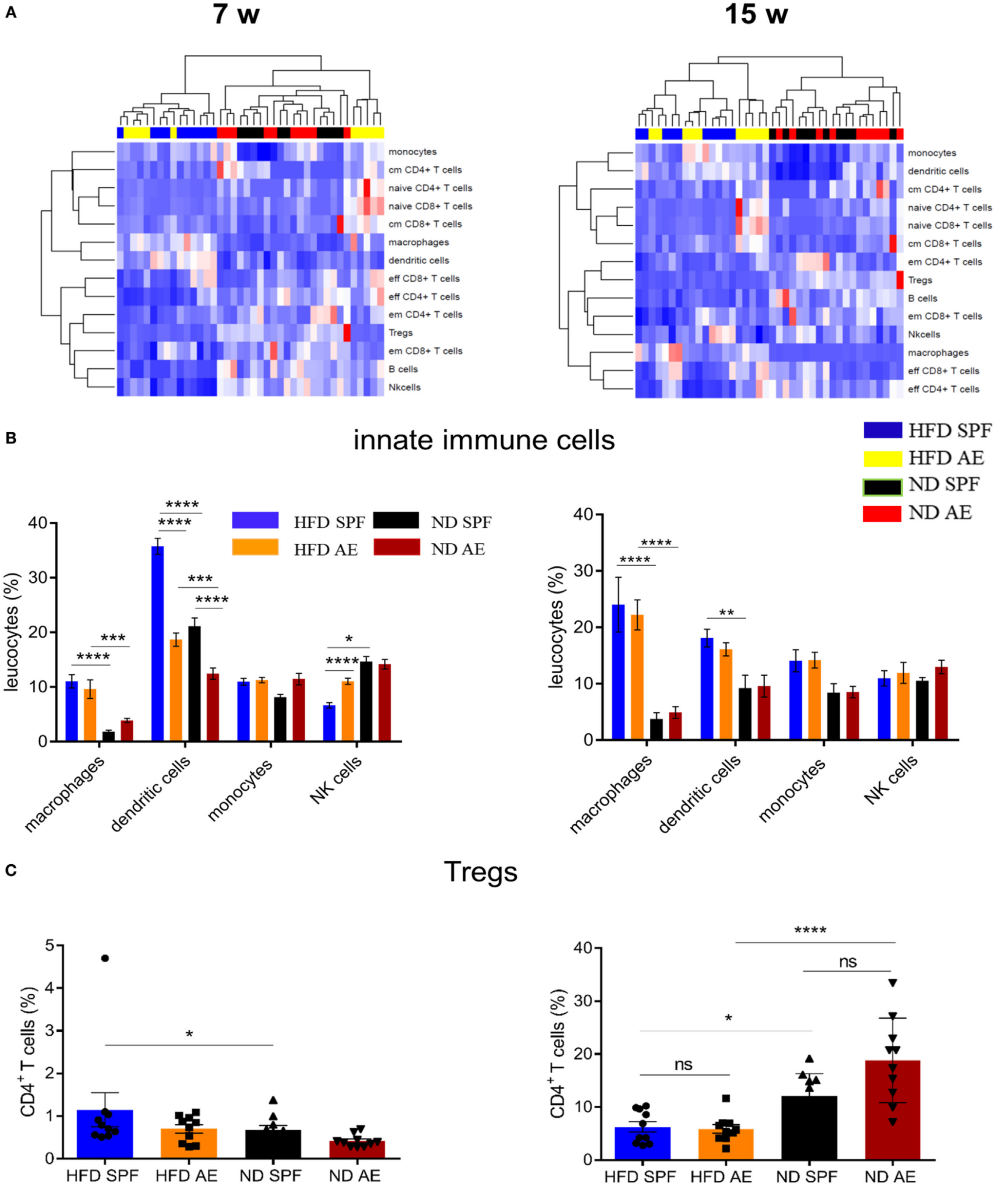

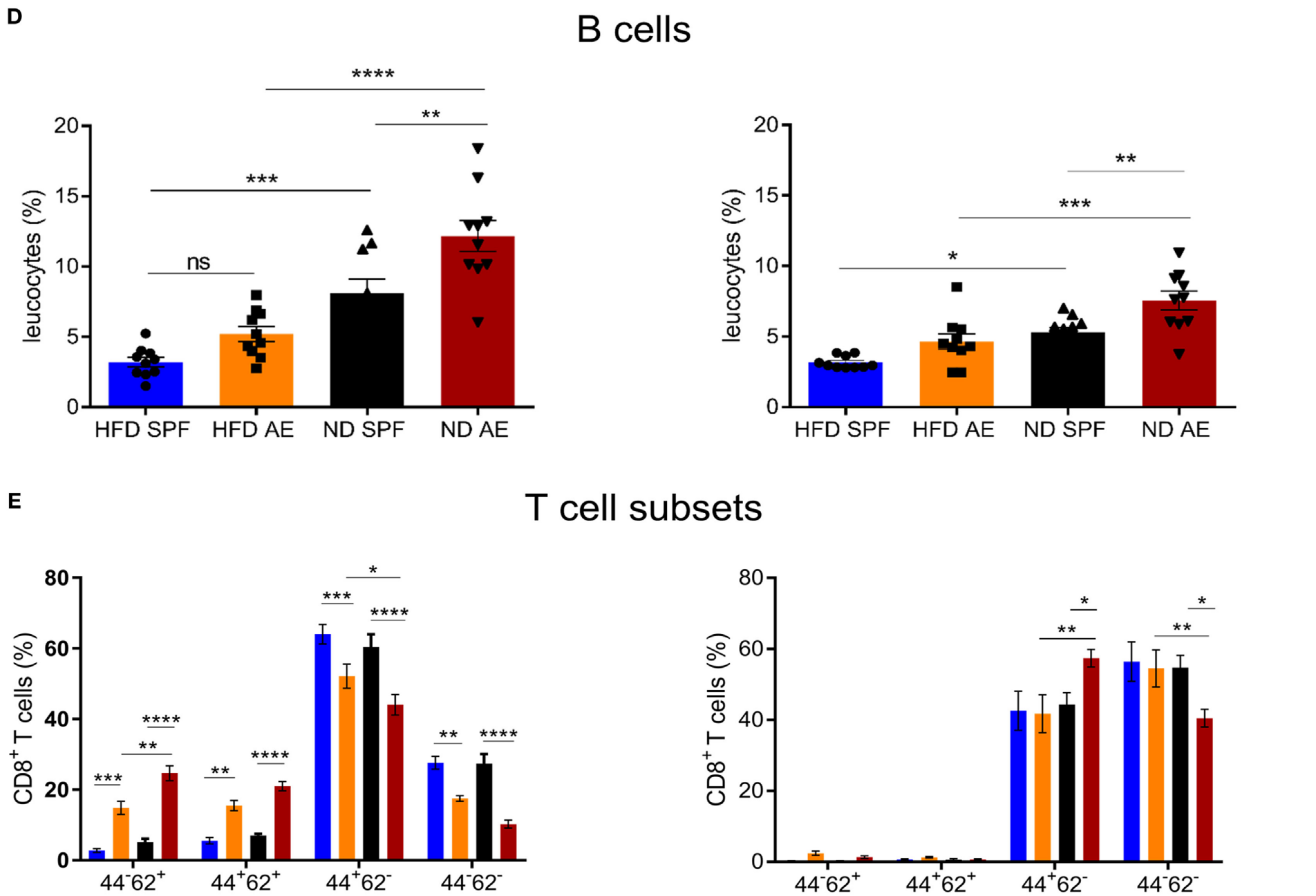
Diet-induced obesity rather than housing conditions change the immune cell composition in visceral adipose tissue (VAT). Immune cell composition was determined in VAT *via* flow cytometry in 7 and 15 weeks fed mice. w, weeks. **(A)** Heatmap showing relative distribution of T cell subsets and cells of the innate immune system as percentages of leukocytes in 7 (left) and 15 (right) weeks groups. Red indicates higher proportion and blue indicates lower proportion. cm, central memory; em, effector memory. **(B)** Distribution of innate immune cells as percentage of leukocytes in high-fat diet (HFD) and normal diet (ND) mice maintained under specific pathogen-free (SPF) or antigen-exposed (AE) conditions in 7 (left) and 15 (right) week groups. **(C,D)** Proportion of regulatory T and B cells in VAT of SPF or AE mice in 7 (left) and 15 (right) week groups. **(E)** Percentages of CD44^−^CD62L^+^ (naïve), CD44^+^CD62L^+^ (central memory), CD44^+^CD62L^+^ (effector memory), and CD44^−^CD62L^−^ (probably effector) T cells in 7 (left) and 15 (right) week groups. Significance was determined using two-way ANOVA multiple measurement **(B,E)** or ANOVA followed by Tukey’s multiple comparisons test **(C,D)** *n* = 10 mice per group. *P < 0.05, **P < 0.01, ***P < 0.001, ****P < 0.0001.

To further investigate the influence of housing conditions and diet on the intra-adipose tissue immune status, we analyzed immune cells in the VAT by flow cytometry. Figure 5A summarizes the relative distribution of various immune cell subsets in HFD AE and SPF mice at week 7 (left) and 15 (right) demonstrating a clear clustering of the mice in regard to diet.

The percentages of macrophages (F4/80^+^CD11b^+^) and DCs (CD11b^+^CD11c^+^) were significantly higher in HFD compared to ND mice, which is in line with previous results (28). However, innate immune cells were equivalent between AE and SPF groups of mice (Figure 5B). Only some temporary differences in dendritic and NK cells were observed between AE and SPF mice after 7 weeks of HFD feeding (Figure 5B). Similarly, both HFD groups showed comparably decreased proportions of regulatory (CD25^high^CD127^−^) CD4^+^ T cells (T_reg_) and B cells (CD3^+^B220^+^) in relation to ND mice (Figures 5C,D). Only after 7 weeks, T_regs_ in HFD SPF mice were higher than in ND SPF mice (Figure 5C). As expected, in all groups (AE vs. SPF; HFD vs. ND) mainly effector/memory T cells accumulated in the adipose tissue (Figure 5E). Interestingly, at week 7, we could also observe naïve-like and central memory T cells in the adipose tissue from AE but not SPF mice (Figure 5E). To further assess the impact of metabolic measures on the systemic immune cell composition, we exploratively examined the correlations between body weight, epigonadal fat weight, HOMA, and leucocyte cell numbers. Significant correlations were found between CD4^+^ and CD8^+^ T cell subsets as shown in Table S2 in Supplementary Material.

In summary, HFD-fed mice housed in SPF or AE conditions display higher levels of distinct innate immune cells compared to ND mice, whereas only temporary and minor differences are observed between SPF and AE mice.

### Antigen Exposure Protects β Cell Morphology and Function Allowing Lasting Compensation of Insulin Resistance

**Figure 6:**
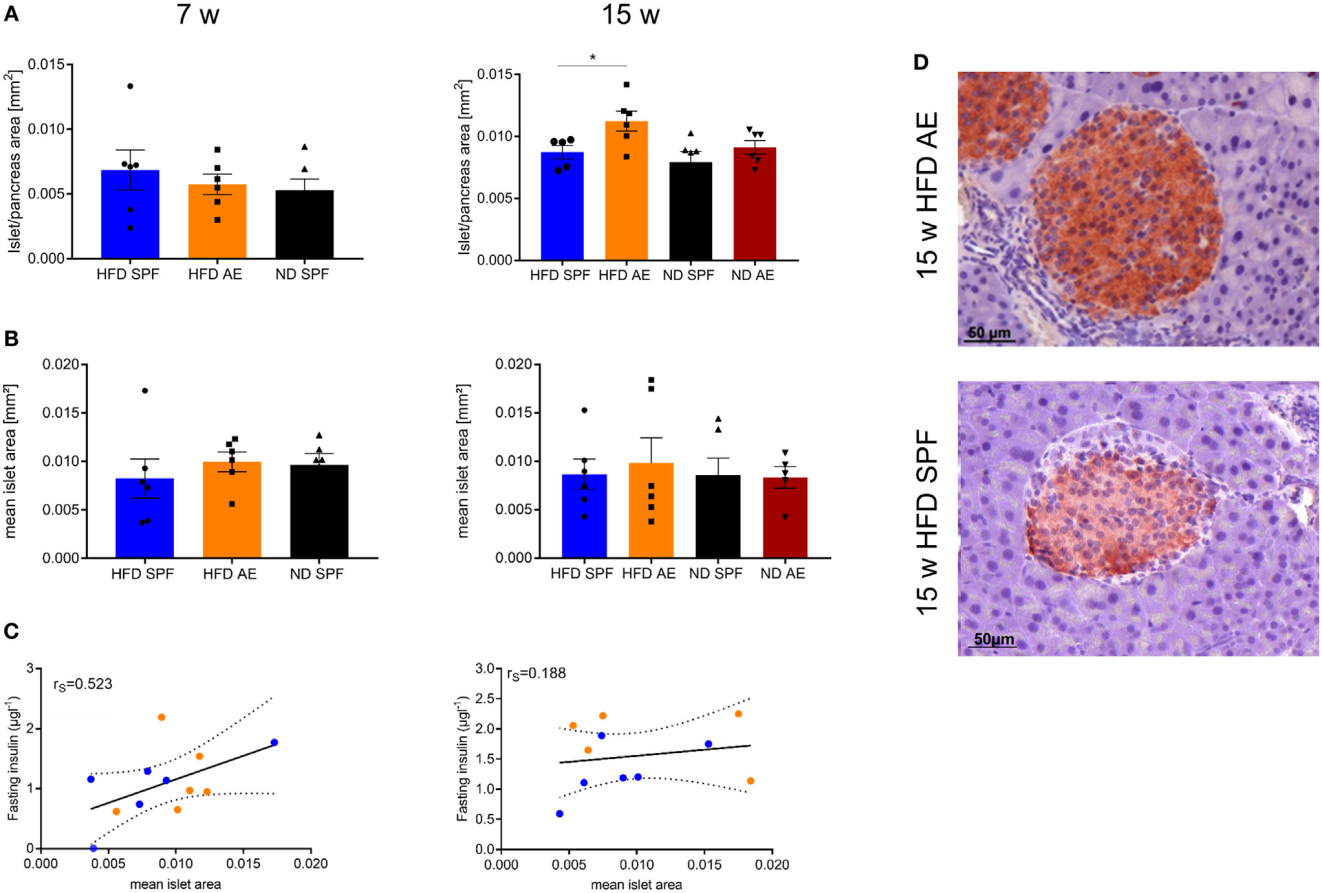
Antigen exposure protects pancreas morphology and function allowing lasting compensation of insulin resistance. Hematoxylin/eosin and immunohistochemical (IHC) insulin stainings were performed in 7 and 15 weeks fed mice. **(A)** Mean islet cross-sectional area expressed as the percentage of the total pancreas area in 7 and 15 weeks fed specific pathogen-free (SPF) and antigen-exposed (AE) mice. Grubbs’ analysis identified one outlier in 15 weeks high-fat diet (HFD)-fed SPF mice. **(B)** Mean islet area in mm^2^ for 7 and 15 weeks fed SPF and AE mice. *n* = 5–6 mice per group. **(C)** Mean islet area initially tends to correlate with fasting insulin in all groups (*P* = 0.0004) (left), but correlation decreases over time because fasting insulin levels are kept high in AE mice only, *P* = 0.011 (right). **(D)** Representative serial sections from mouse pancreas immunostained for insulin. Results are representative of five to six mice. Significance was determined using unpaired two-sided Mann–Whitney *U*-test or one-way ANOVA. Scale bar, 50 µm.

Next, we examined the histology of the pancreas *via* HE, IHC insulin, and T cell staining to understand the metabolic differences between the groups. In comparison to ND mice, HFD mice developed a small degree of islet hyperplasia as described before (29). Furthermore, HFD AE mice showed a higher mean cross-sectional area of the pancreatic islets at week 15 compared to HFD SPF mice (Figure 6A). Mean islet area was comparable in mice under SPF conditions, whereas HFD AE mice tended to display a higher mean islet area compared to ND AE mice (Figure 6B). However, higher plasma insulin levels did not correlate significantly with the mean islet area (Figure 6C) of 7 and 15 weeks HFD mice in both the SPF and AE group. In the HFD SPF group, grossly diminished numbers of β cells were evident, while HFD AE mice showed preserved insulin staining (Figure 6D). Notably, periductal pancreatic inflammation was neither observed in SPF nor in AE mice. Moreover, immunostaining revealed that almost all CD3^+^ cells remain in the vessels without any alterations between groups (data not shown).

To sum up, histological analyses of pancreatic islets confirm increased β cell compensation in AE mice that persisted over the observation time of 15 weeks HFD feeding.

### HFD in Immune-Aged Mice Is a High Risk Combination for the Development of Non-Alcoholic Steatohepatitis (NASH)

**Figure 7:**
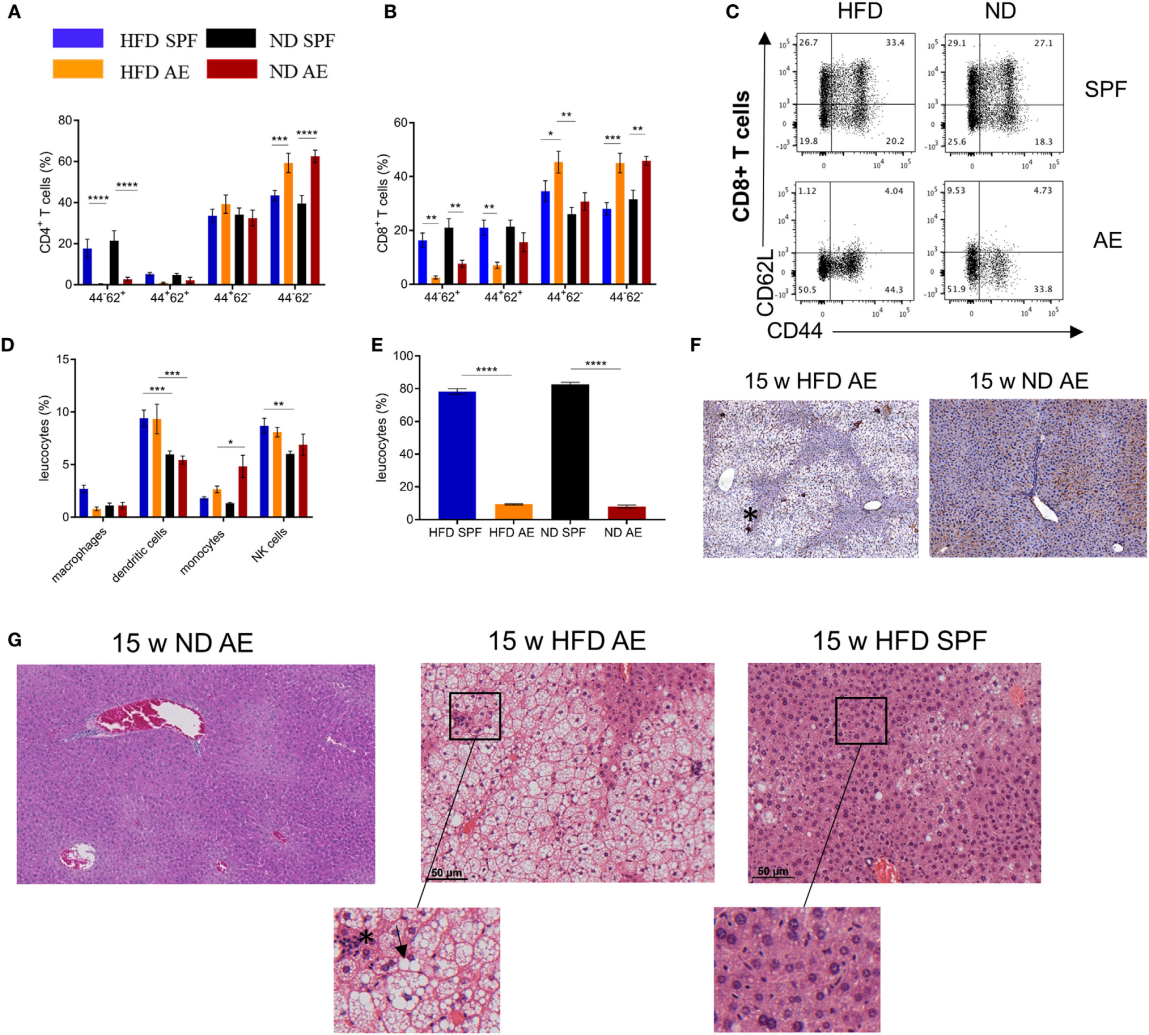

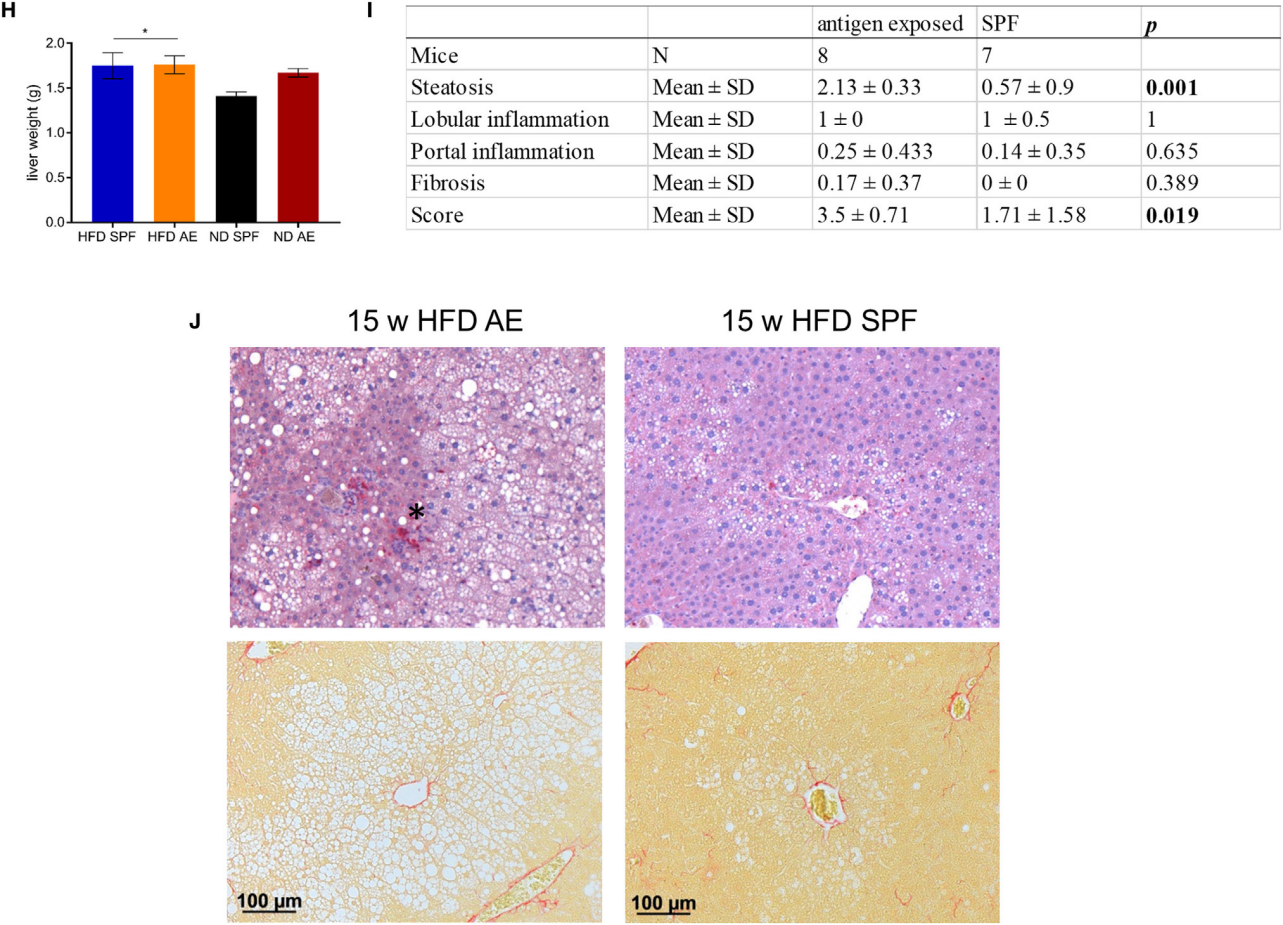
High-fat diet (HFD) in immune-aged mice is a high-risk combination for the development of non-alcoholic steatohepatitis (NASH). Immune cell composition in livers of 7 weeks fed specific pathogen-free (SPF) and antigen-exposed (AE) mice was analyzed *via* flow cytometry. **(A–C)** CD4^+^ and CD8^+^ T-cell liver phenotypes were compared among 7-week HFD and normal diet (ND) mice maintained under SPF and AE conditions. **(D)** Percentages of innate immune cells in liver tissue. MHCII staining was performed in 15 weeks fed AE mice. **(E,F)** Percentages of immature (MHC^low^) dendritic cells in liver tissue and representative Immunohistochemical (IHC) staining with MHCII (asterisk) in 15-week HFD and ND AE mice. Hematoxylin/eosin stainings were performed in 15 weeks fed SPF and AE mice. **(G)** Representative staining of ND AE and HFD SPF and AE mice. Infiltration of immune cells (asterisk) and ballooned hepatocytes (arrowhead) illustrate NASH. *n* = 5–10 mice per group. **(H)** Liver weight of 15 weeks fed mice, *n* = 10. Significance was determined using one-way ANOVA *P < 0.05. **(I)** Table showing histological scoring system for NASH. **(J)** IHC staining of CD3-expressing T cells (upper row) and Sirius Red staining (lower row) in paraffin-embedded liver sections from mice fed an HFD or ND for 15 weeks. Asterisk indicates CD3 staining, *n* = 6–10 mice. Significance was determined using two-way ANOVA multiple measurement. *P < 0.05, **P < 0.01, ***P < 0.001, ****P < 0.0001.

Obesity is frequently associated with liver steatosis that can progress to NASH including a negative predictive value for the course of T2D and metabolic syndrome. Therefore, we next asked, whether housing conditions would have an impact on liver pathology. Intrahepatic naïve CD4^+^ and CD8^+^ T cells were found to be considerably lower in AE compared to SPF mice at week 7 (Figures 7A,B). Conversely, for CD8^+^ T cells, the fraction of effector memory T cells was significantly higher (Figure 7B), which was most pronounced in AE mice on HFD. Notably, more than 95 and 85% of CD4^+^ and CD8^+^ T cells, respectively, expressed the effector memory T cell phenotype in AE mice on HFD, a delta of >25% compared to SPF mice on HFD (Figures 7A–C). The innate cell subsets were less dramatically changed with trends for partial replacement of macrophages by infiltrating monocytes in AE mice and a slightly enhanced frequency of NK cells in both HFD groups (Figure 7D). In addition, SPF mice showed lower percentages of immature (MHCII_low_) DCs (Figure 7E) and HFD mice displayed higher MHCII antigen expression levels under SPF conditions (Figure 7F).

Hematoxylin/eosin staining of liver sections of mice on HFD for 15 weeks revealed severe steatosis in AE mice while only some SPF mice displayed a mild fat accumulation in the liver. In addition to a strong increase of large lipid droplets resulting in macrovesicular steatosis, the livers of AE mice on HFD showed lobular inflammation, hepatocyte injury in the form of hepatocellular ballooning, and destroyed lobule structure. However, livers of ND AE mice did not display any signs of NASH (Figure 7G). However, liver weight did not differ between all groups (Figure 7H). Blinded NASH scoring of liver histology confirmed that 8/8 AE mice on 15 weeks HFD displayed a strong NASH phenotype (score > 3–5), whereas NASH was not observed in the majority of SPF mice (5/8, score < 1) (Figure 7I). The remaining liver sections were categorized as displaying only a mild form of NASH (3/8, score = 3) (Figure 7I). Furthermore, CD3 staining confirmed lobular inflammation in HFD AE mice (Figure 7J, upper row), whereas Sirius Red staining revealed mild pericellular fibrosis in those mice (Figure 7J, lower row).

Altogether, AE mice display protected insulin production from pancreatic islet cells but develop more severe hepatic steatosis.

## Discussion

We confirm our hypothesis that “aging” of the adaptive immune system has a major impact on the course of obesity-related insulin resistance. Whereas obesity-induced insulin resistance is rather comparable in both groups fed a HFD, AE mice showed improved glucose tolerance by preserved compensatory β-cell function (protective effect). By contrast, immune aging was associated with rapid development of NASH (detrimental effect). The data underline the need for novel preclinical models that are closer to the human situation.

The prevalence of obesity and T2D is increasing worldwide (30). It is widely accepted that adipose tissue inflammation contributes to the development of obesity-related insulin resistance (31, 32). Various components of both the innate and the adaptive immune systems were identified as major players in regulating inflammatory processes in the development of insulin resistance (33–35), vasculitis, and remodeling of parenchymal organs (36–38). However, knowledge about the exact pathomechanisms driving the process across the checkpoints is limited. Recently, awareness arose about the limitations of commonly used animal models regarding the shortcomings of clinical challenges (39, 40). With increasing knowledge about the role of the adaptive immune system, in particular of tissue-resident and tissue-infiltrating T cells, in controlling tissue homeostasis it is more evident than ever that our widely used SPF mouse models do not reflect the “aging” immune system as seen in adults (23, 24).

Abnormally hygienic housing conditions of laboratory mice may account for limited translational potential to humans. To tackle this issue, we applied our recently developed model of AE housing and compared the development of insulin resistance, glucose tolerance, β-cell function, and liver histology in two HFD-fed groups: one kept SPF and the other outside the SPF barrier in AE conditions.

Consistently, AE mice expressed a higher proportion of antigen experienced CD4^+^ and CD8^+^ T cells. In particular, memory effector T cell subsets were increased. Notably, AE mice did not acquire infectious diseases, as monitored following FELASA guidelines on a regular basis.

Our results demonstrate that changes in the immune cell composition of HFD-fed C57BL/6J mice affect the time course of glucose intolerance and insulin secretion. Interestingly, in contrast to our hypothesis, development of obesity-induced insulin resistance was comparable between the two HFD groups. This is in line with immune cell subsets in VAT. Even in SPF mice, we observed a dominance of effector/memory T cells in VAT. Remarkably, HFD in both groups (SPF and AE) enhanced the effector/regulatory T cell ratio resulting in an immune disbalance in adipose tissue. We already mentioned that hypoxia associated with adipocyte hypertrophy can lead to the activation of NFκB-dependent pro-inflammatory responses in adipocytes (6, 7). The increase of systemic chronic inflammation by antigen exposure could further contribute to this hypoxia-dependent pathomechanism and increase the activation of pro-inflammatory cytokines in turn.

Intriguingly, AE mice fed a HFD revealed higher β-cell responsiveness that was observed as excess in insulin levels, which compensates for glucose intolerance for at least 7 weeks. Furthermore, hyperinsulinemia in AE mice was in accordance with elevated body weight compared to SPF mice. As expected, pancreas histology revealed enlarged islet areas that correlated initially with fasting insulin levels in HFD mice. By contrast, β cells in SPF mice lost their functionality over time, confirmed by decreased insulin secretion and the strong correlation between islet mass and fasting insulin levels. Remarkably, β cells from AE mice were protected allowing continuous hyperinsulinemia. So far, the mechanism behind the protective effect is unclear. The number of pancreas infiltrating T cells was roughly equal in both groups. Most T cells stuck to the perivascular region and did not enter the islets. As the pancreas expresses many IL-22 receptors (41), it could be speculated that Th17/22 cells might enter to secrete the pro-regenerative, islet-protective cytokine IL-22.

An alternative explanation might be that a vagus and/or sympathetic activation caused by systemic and local inflammation triggers the so-called “anti-inflammatory reflex” in pancreatic islets in AE mice, as described recently in critically ill patients suffering from major surgery or stroke-induced immunodeficiency (42–44).

Linked to the increasing prevalence of obesity worldwide, NASH, the more inflammatory and progressive form of non-alcoholic fatty liver disease, emerges as a major health burden in developed countries (45). NASH is histologically characterized by ballooned hepatocytes, lipid accumulation, fibrosis, and pericellular inflammation and may progress to cirrhosis, end stage liver disease, or hepatocellular carcinoma (46). Several diets are known to induce NASH-like liver pathology in C57BL/6J mice (47), but most of these relatively artificial approaches do not recapitulate human conditions of NASH and its metabolic consequences. Here, we show for the first time that mice maintained under non-SPF “antigen exposed” conditions develop NASH-like liver pathology as early as after 7 weeks of HFD feeding. They developed macrovesicular steatosis, hepatic infiltration, and altered intrahepatic immune cell composition rarely seen in SPF mice even after 15 weeks on HFD. Recently, it has been described (48) that metabolic activation of intrahepatic immune cells causes NASH in C57BL/6J mice in concordance with our results. Thus, our study offers a simple mouse model of short-term HFD for investigating the development of NASH and the underlying mechanisms that also exhibits fidelity to the human condition.

Based on our results, a number of questions arise. First, future studies are required to investigate the exact mechanisms of T cell activation in the liver and to tackle the question whether a systemic immune activation, induced by diet and housing conditions, or the dissemination of T cells activated in the liver accounts for NASH-like pathology and liver function. In contrast to these findings, severe liver inflammation was not accompanied by pancreatic tissue inflammation and did not correspond to β-cell compensation for insulin resistance. Therefore, further studies have to be performed to validate the mechanisms of β cell protection, which are currently unknown. Similarly, in patients, severe steatohepatitis is often not attended by T2D, even though it is assumed that NASH is associated with insulin resistance (49). Second, the composition, diversity, and function of the gut microbiome was described to be affected by HFDs (50–52), revealing the phenotypes seen in AE mice in a different light. Recently, it has been published that gut barrier function, e.g., intestinal permeability and alterations in intestinal levels of secondary bile acids, depend on housing conditions (53). The question whether gut colonization plays a causal role in adipose tissue and liver inflammation will be addressed in a forthcoming publication. Finally, the influence of food and nutrients composition should be considered.

In conclusion, our study identifies an important new immunological link between environmental conditions and the progression of obesity, insulin resistance, and its comorbidities. We provide a new mouse model valuable for revealing new biomarkers of metabolic disease progression as well as testing novel therapeutic approaches.

## Availability of Data

The datasets generated during and/or analyzed during the current study are available from the corresponding author on reasonable request.

## Ethics Statement

This study was carried out in accordance with the Guide for the Care and Use of Laboratory Animals of the National Institutes of Health and the Animal Welfare Act under the supervision of our institutional Animal Care and Use Committee. Animal protocols were conducted according to institutional ethical guidelines of the Charité Berlin, Germany, and were approved by the Landesamt für Gesundheit und Soziales (approval number G 0138/14, LAGeSo Berlin, Germany) and comply with the ARRIVE guidelines. This study was carried out in accordance with the recommendations of the International Conference on Harmonization Guidelines for Good Clinical Practice and the Declaration of Helsinki. The protocol of the study was approved by the local Ethics Committee of the Charité – Universitätsmedizin Berlin (EA2/050/10). All subjects gave written informed consent in accordance with the Declaration of Helsinki before participating in this study.

## Author Contributions

JS-K planned the study, wrote the manuscript, performed experiments, and analyzed data. JK did immunostaining of liver and pancreas sections. SB helped with metabolic experiments and carefully reviewed the manuscript. MS contributed to the selection of antibody panels and provided help with flow cytometry analysis. MH contributed to conception and design of the study. AK performed CD3 staining of pancreas sections. KS-B contributed to the invention of the AE mouse model. KM planned and carried out the clinical study “Effects of negative energy balance on muscle mass regulation” (registered at https://clinicaltrials.gov, NCT01105143). JS planned the concept of the study as endocrinological supervisor and reviewed the manuscript. H-DV planned the concept of the study as immunological supervisor and wrote parts of the manuscript. JS-K, JS, and H-DV are the guarantors of this work and, as such, had full access to all the data in the study and take responsibility for the integrity of the data and the accuracy of the data analysis.

## Conflict of Interest Statement

The authors declare that the research was conducted in the absence of any commercial or financial relationships that could be construed as a potential conflict of interest.
