# Supplementary material for: Distinct Housing Conditions Reveal a Major Impact of Adaptive Immunity on the Course of Obesity-Induced Type 2 Diabetes
Source: Front Immunol. 2018 May 28;9:1069. doi: 10.3389/fimmu.2018.01069 (PMC5985496; doi:10.3389/fimmu.2018.01069)
Supplement: Supplementary file 1 [file data_sheet_1.docx]

**Supplementary Materials**

**
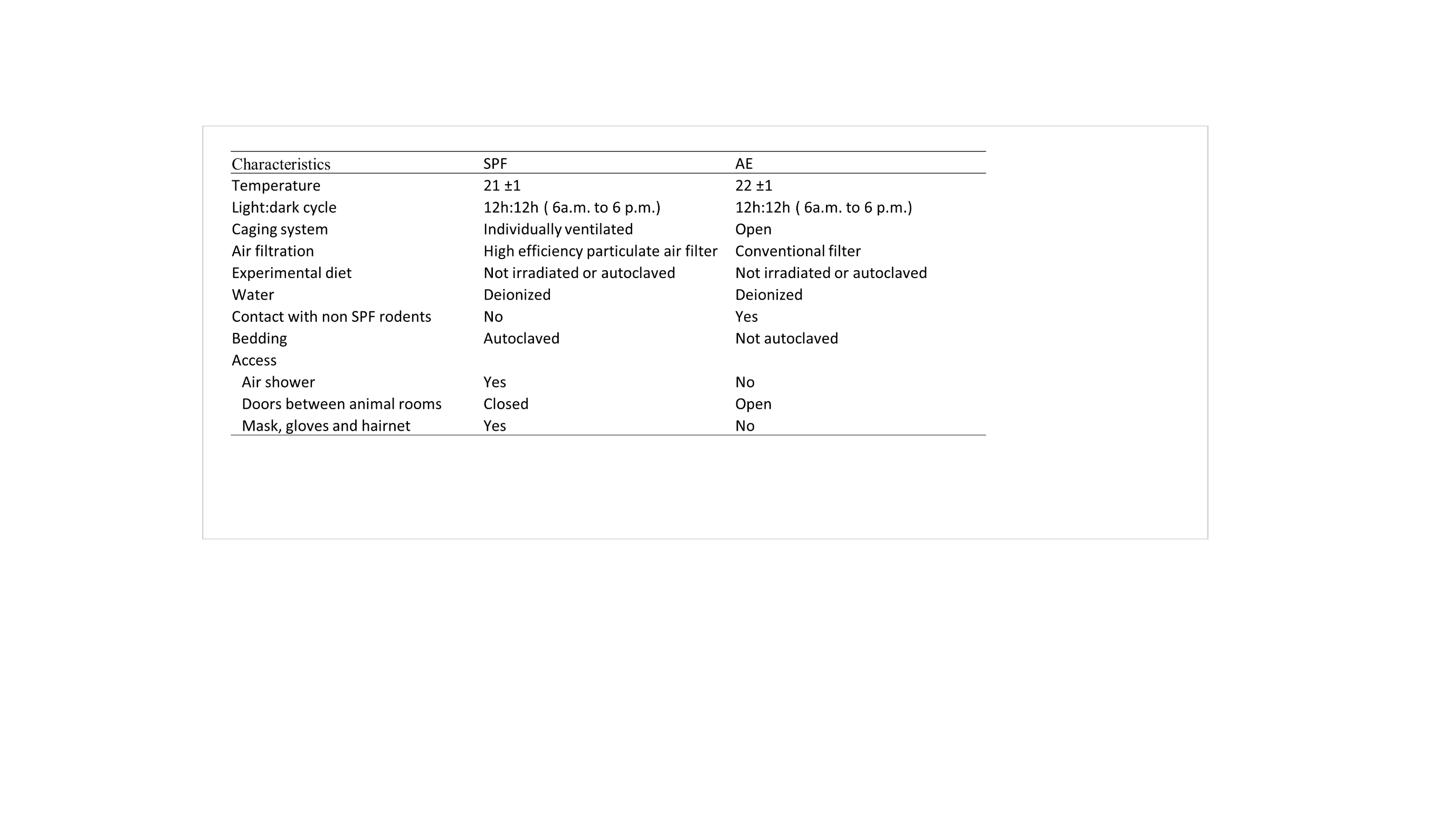
Supplementary Table 1.** Housing conditions in SPF and AE facilities. Animals in both facilities were monitored following FELASA guidelines.


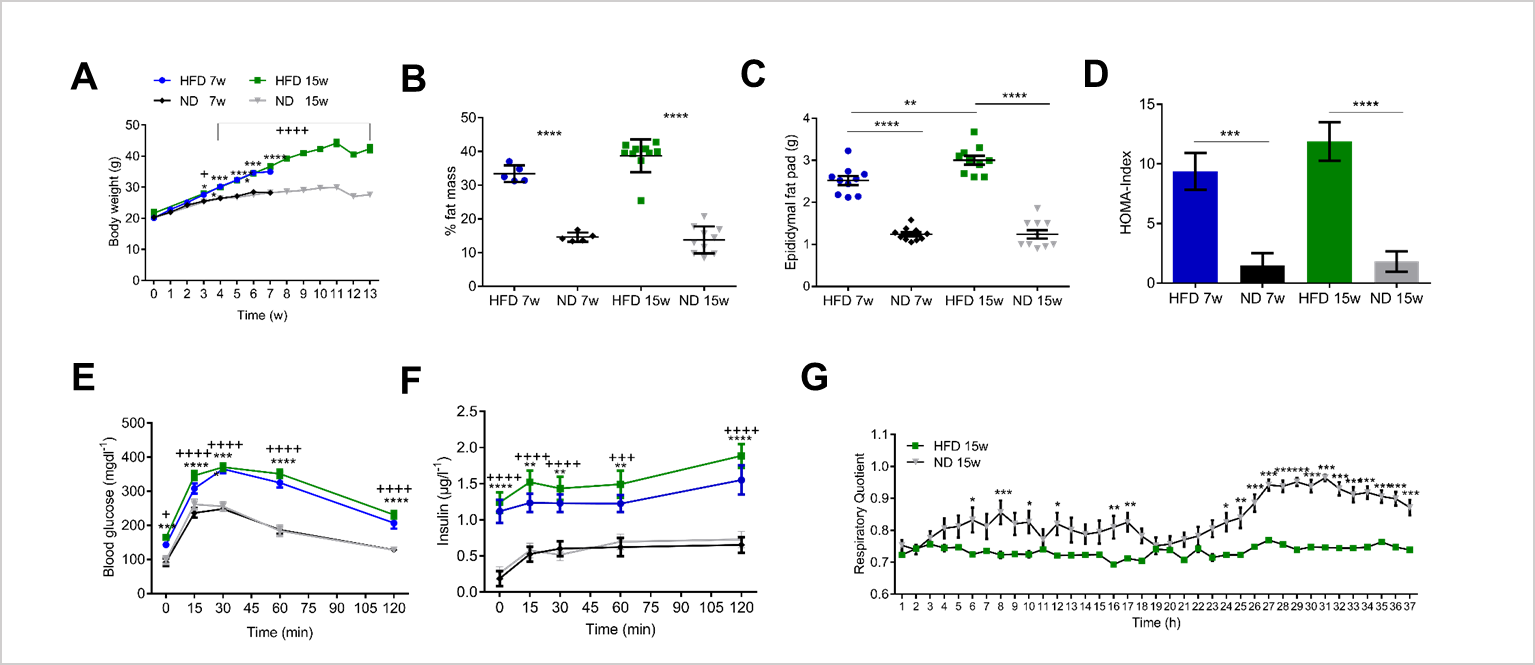


**Supplementary Figure 1.** Wildtype SPF mice fed a HFD for 7 or 15 weeks develop glucose intolerance and insulin resistance. (A) Weight development in male 5 week-old C57BL/6 mice (n=10 per group) upon 7 or 15 weeks of HFD compared to mice fed ND. (B), (C) Quantitative analysis of the body composition by MRI and calculated weight of epididymal fat pads in g. (D) HOMA-index calculated as fasting plasma insulin (in milliunits per liter) × fasting plasma glucose (in mg per deciliter)/450. (E), (F) Blood glucose and insulin levels in an IPGTT performed with 6 weeks and 12 weeks fed mice. (G) Respiratory Quotient calculated for 11 weeks fed mice during a 48h lasting observation period in metabolic cages. Significance was determined using 2-way ANOVA multiple measurement test. *P<0.05 (HFD 7week vs. ND 7week), +P<0.05 (HFD 15week vs. ND 15week), **P<0.01 ((HFD 7week vs. ND 7week), ++P<0.01 (HFD 15week vs. ND 15week), ***P<0.001 (HFD 7week vs. ND 7week), +++P<0.001 (HFD 15week vs. ND 15week), ****P<0.0001 (HFD 7week vs. ND 7week), ++++P<0.0001 (HFD 15week vs. ND 15week). w=week.

**
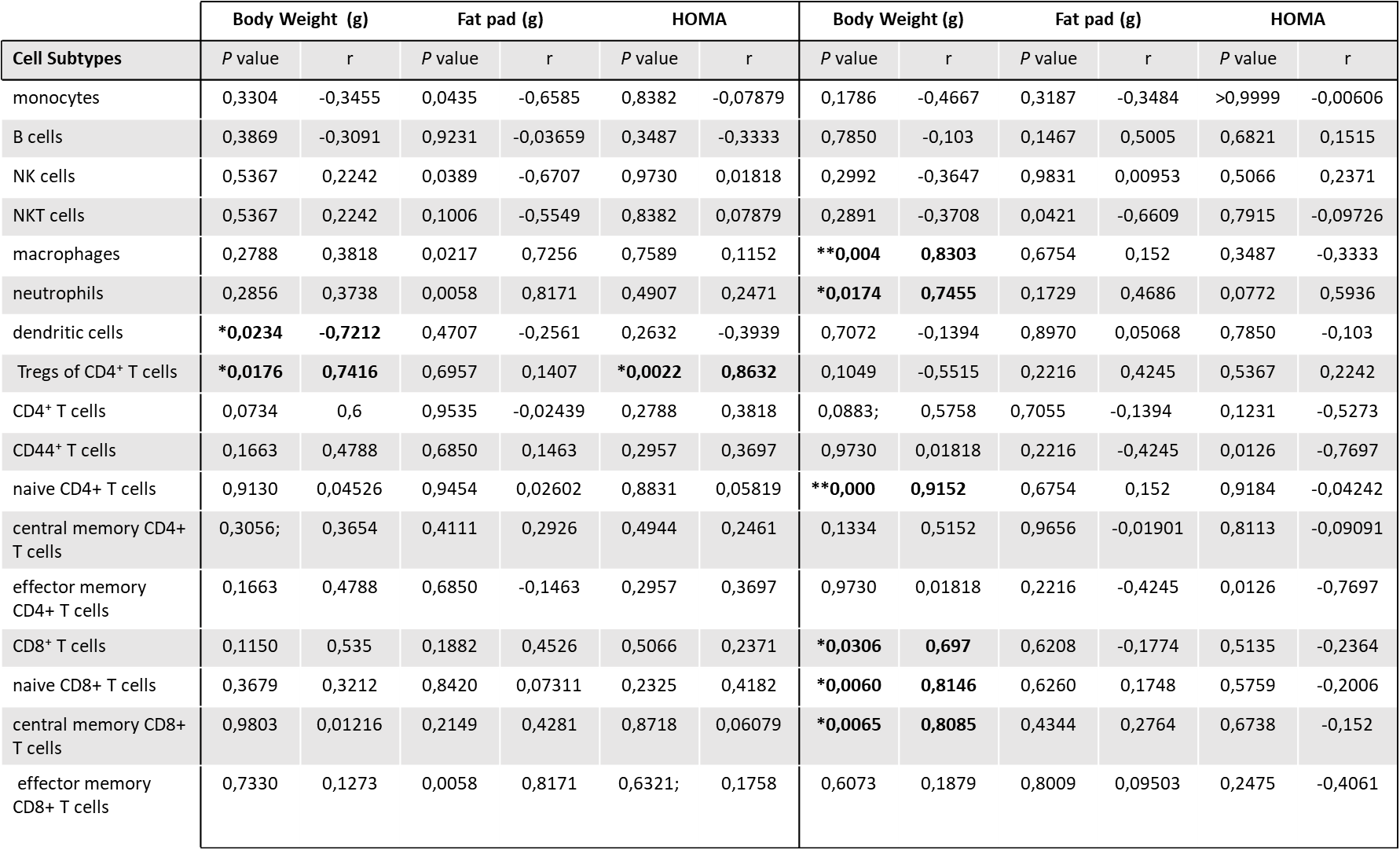
**

**Supplementary Table 2.** Metabolic measurements correlate with immune cell subtypes in visceral adipose tissue of 15 weeks fed HFD SPF (left column) and AE (right column) mice. Significance was determined applying Spearman Correlation with 95% confidence interval. *P<0.05, **P<0.01.

**
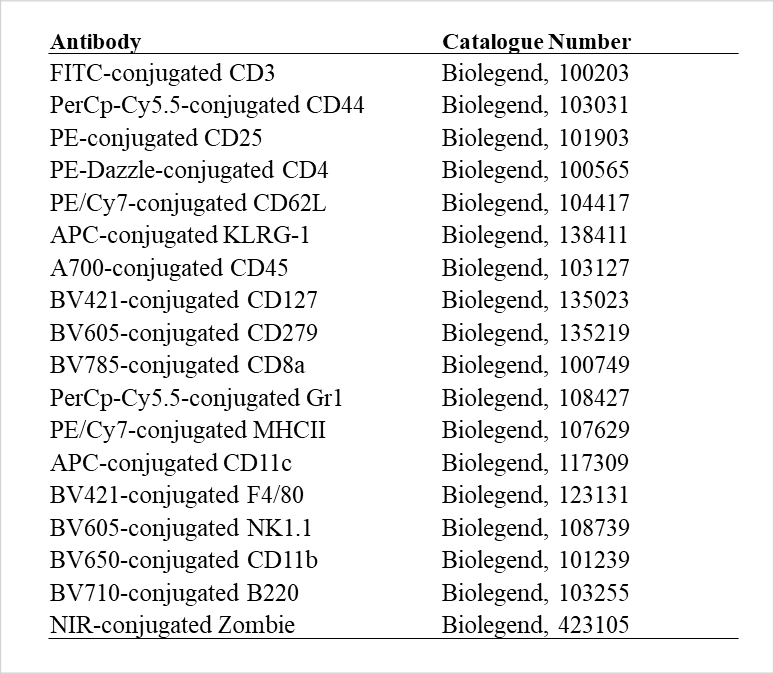
**

**Supplementary Table 3.** Antibodies used in flow cytometry staining. NIR-conjugated Zombie was used as live/dead marker.
